# The Role of High-Fructose Diet in Liver Function of Rodent Models: A Systematic Review of Molecular Analysis

**DOI:** 10.52547/ibj.3965

**Published:** 2023-07-29

**Affiliations:** 1 Department of Toxicology and Pharmacology, Faculty of Pharmacy and Pharmaceutical Sciences, Tehran Medical Sciences, Islamic Azad University, Tehran, Iran;; 2 Food and Drug Laboratory Research Center, Food and Drug Administration, MOH&ME, Tehran, Iran

**Keywords:** Animal model, Fructose, Liver, Liver function tests, Oxidative stress

## Abstract

The present systematic review of animal studies on long-term fructose intake in rodents revealed a significant decrease in the activities of antioxidant enzymes due to a fructose-rich diet. The reduced activity of these enzymes led to an increase in oxidative stress, which can cause liver damage in rodents. Of eight studies analyzed, 5 (62.5%) and 1 (12.5%) used male and female rats, respectively, while 2 studies (25%) used female mice. Moreover, half of the studies used HFCS, but the other half employed fructose in the diet. Hence, it is essential to monitor dietary habits to ensure public health and nutrition research outcomes.

## INTRODUCTION

Fructose is naturally found in honey and sweet fruits, although for commercial purposes, it is typically produced in crystalline form or as syrup. Corn syrup is a common commercial product of fructose that has increasingly become popular in recent years and contains around 55% of free fructose. Over the past two decades, the use of HFCS as a sweetener in beverages and processed foods has raised by 20-30%^[^^1^^,^^2^^]^. 

Hyperglycemia and insulin resistance are two risk factors for T2DM^[^^3^^]^. This disorder can cause various hepatic injuries and abnormalities, including hepatitis, NAFLD, nonalcoholic steatohepatitis, cirrhosis, and hepatocellular carcinoma^[^^4^^,^^5^^]^. The increased use of artificial sweeteners such as fructose and HFCS in processed foods might be related to the occurrence of these disorders.

Multiple preclinical and clinical studies have suggested a potential link between high fructose consumption and metabolic syndrome^[^^6^^]^. It has also been demonstrated that excessive consumption of fructose by healthy individuals over a short period of time can predispose the individuals to be overweight. The harmful effects of fructose on health are attributed to how it is metabolized when consumed in food^[^^1^^,^^6^^,^^7^^]^. High fructose intake has been associated with fatty liver disease, although the underlying mechanisms are not yet fully understood^[^^8^^]^. Interestingly, a study has found that high-fructose diets can lead to significant effects and changes in various liver metabolism pathways^[^^1^^]^. Apart from alterations in the transfer function of fatty acids, VLDL-TG assembly, cholesterol metabolism, and multiple molecular changes have been identified due to high-fructose diets. These changes include modifications in molecular chaperones involved in protein folding in the ER, and alteration in the protective activity of antioxidant. It has been observed that the high intake of fructose can have an adverse effect on liver metabolism, especially in the context of liver enzyme activity^[^^1^^,^^9^^]^. The present study suggests a molecular-based approach that explains metabolic changes resulting from high-fructose diet.

Recent epidemiological studies have demonstrated a strong link between intrahepatic fat accumulation and saturated fats, carbohydrates, simple sugars, and trans fatty acids^[^^10^^]^. However, further investigation is needed to fully understand relationship between specific nutrients containing sugars and specific nutrients containing fructose. From hepatology perspective, understanding the molecular effects of long-term fructose consumption on liver injury is of high importance. While rodent models have extensively been used in research and contributed immensely to the understanding of human diseases, there are still some uncertainties and limitations that need consideration. The authors of this study aimed to provide a comprehensive review of the outcomes of liver damage caused by high-fructose consumption. This survey could help to better understand the impact of high-fructose consumption on liver health and inform future research and potential strategies to prevent or treat liver damage. By better understanding the underlying molecular mechanisms, researchers can develop more effective treatments for patients suffering from liver-related illnesses.

**METHODOLOGY**

The present systematic review was conducted based on the PRISMA. Literature searches were carried out by two independent reviewers (from 1985 to June 2023) in four international databases, comprising Web of Science, PubMed, Embase, and Scopus. In searches, we used the MESH terms and other related keywords shown in Supplementary Table 1.

**Inclusion and exclusion criteria**

Two independent reviewers were responsible for the selection of all animal studies. In case of disagreement, a third reviewer was consulted. By a refined search strategy, we removed duplicate studies, and by further screening, we eliminated irrelevant studies after assessing their titles, abstracts, and full texts. It is important to know that mice share many anatomical, physiological and genetic similarities with humans. The use of rodent models in preclinical research has played a vital role in advancing our comprehension of human diseases, allowing researchers to explore underlying mechanisms and potential therapies that may not be feasible to directly study on humans. By conducting genetic research on mice, scientists gain insights into the biology of human disease and develop safe and effective treatments. Development of molecular biology has enabled researchers to extrapolate insights from rodent research and understand the molecular changes that cause liver injuries, paving the way for a better understanding of liver metabolism pathways and how they respond to different diets, including high-fructose diets. Following the investigation of underlying molecular mechanisms, they also can gain a better understanding of the pathogenesis of these diseases, which assists them to develop new treatments targeting specific molecular pathways, thereby revolutionizing the treatment of liver diseases. The inclusion criteria for this study were quite specific and limited to rodents that were exposed to either HFCS or a high-fructose diet, without any mixture. This review excluded studies in which species other than rodents were used. We also excluded a variety of research types, such as case studies, case series, reviews, editorials, conference reports, letters, human and in vitro studies, and epidemiological studies, which assessed the effects of on toxicity rather than liver toxicity.

**Data extraction**

Data were extracted based on a pre-defined checklist. The extracted data used for qualitative evaluation included the date and topics of study, author's name, sample size of the groups, information about the animals (species and genus), amount and routes of administration, age and gender of the rodents, duration of the study, methods of assessment, molecular findings, and the main findings of each study. A summary of the data extraction checklist is shown in the Table 1.

**Quality assessment**

The Joanna Briggs Institute Critical Appraisal tools was employed to evaluate the quality of the selected articles. Only studies answered yes to at least 60% of the questions on the specific checklist were included. This assessment ensured that we selected only high-quality articles for our systematic review. The authors carefully evaluated the quality of all articles and resolved discrepancies or disagreements by discussion. 

## RESULTS

**Study selection**

**Fig. 1:**
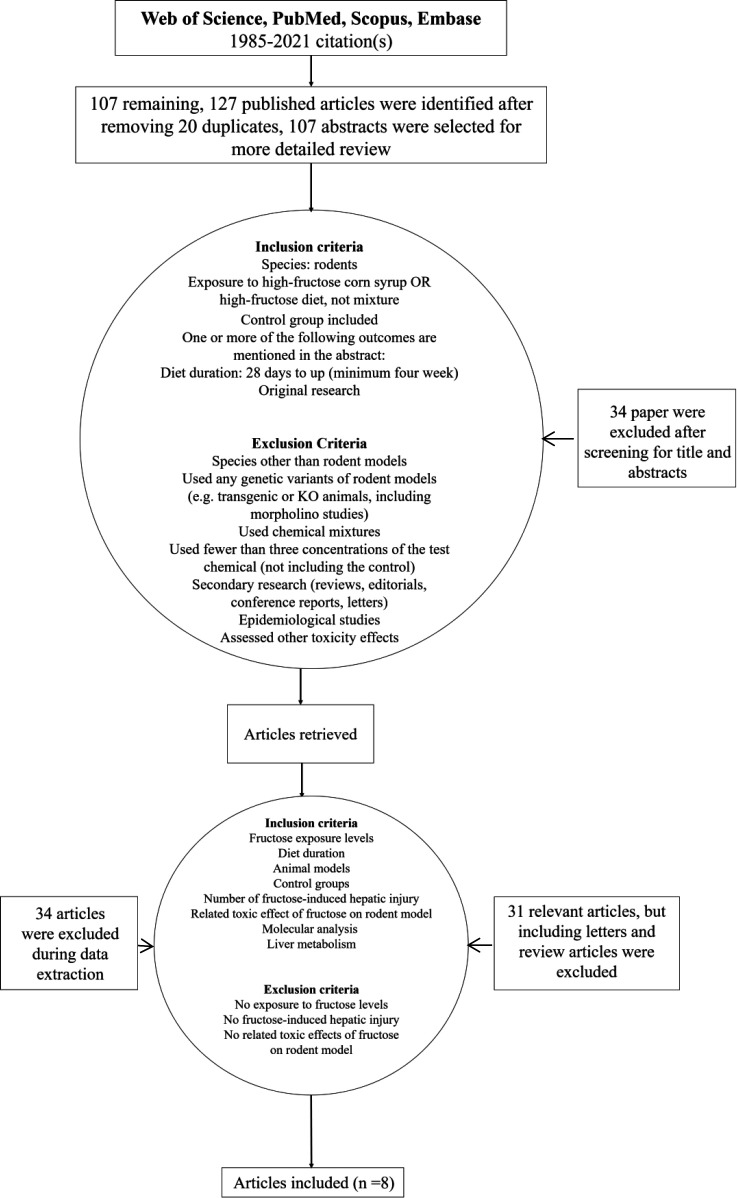
Flow Diagram of study selection

**Fig. 2:**
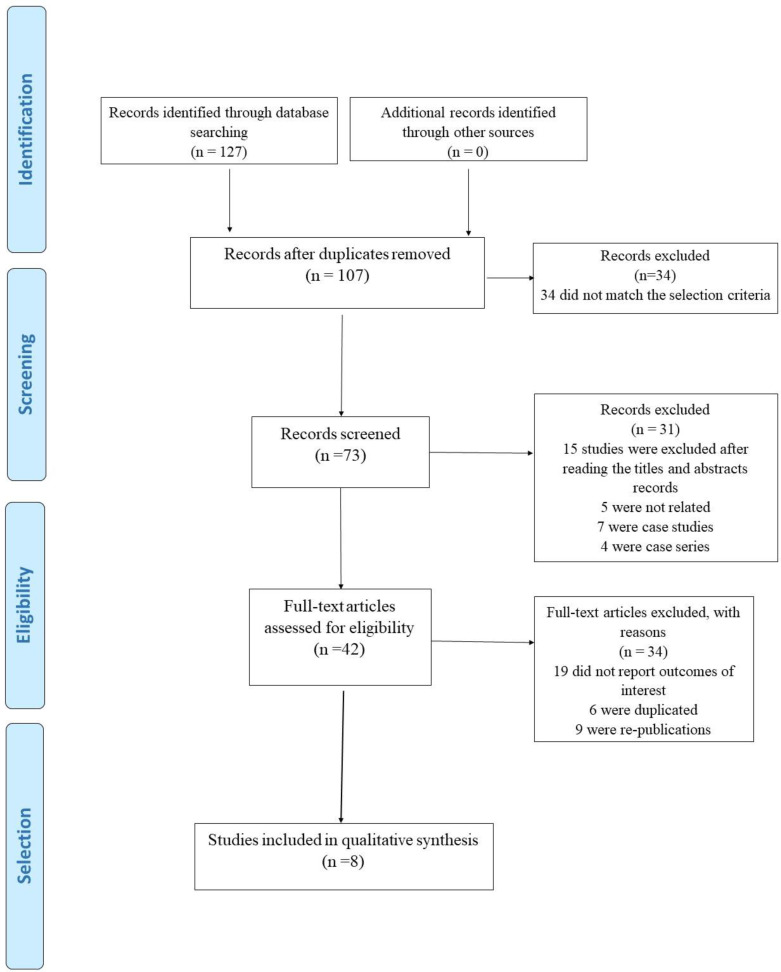
PRISMA flowchart for included studies

Following our search strategy, 127 published articles were identified. After removing 20 duplicates, 107 abstracts were selected for a more detailed review; 34 papers were excluded after screening for title and abstracts and 31 articles, including letters and review articles, were also removed. Eight articles were finally selected from the remaining 42 relevant papers for the current systematic review (Figs. 1 and 2).

**Table 1:** Summary of data extraction checklist

**Authors/Date**	**Country**	**Evaluation of exposure and outcome**	**Molecular analysis/gene expression**	**Duration of study**	**Administration routes **	**Amount of fructose/** **HFCS**	**Species /gender of animals**		**Control group**		**Number of test group **			**Topics**	**No.**	
**Cioffi ** **2017** ^[^^11^^]^	Italy	Mitochondrial DNA damage A fructose-rich diet leads to mitochondrial damage (mtDNA), which may play a role in liver dysfunction and metabolic disease.	Yes	8 weeks	Oral- drinking water	HFCS: 20% solution	Male rats (Sprague Dawley)		Yes		Two groups (five per group)			Fructose-rich diet affects mtDNA damage and repair in rats	1	
Taleb-Dida 2011^[^^12^^]^	Algeria	Hypertriglyceridemia High-fructose intake in the diet causes hypertriglyceridemia.	Yes	14 weeks	Oral	61(g/100 g)	Male rats (Wistar)		Yes		Four groups (six per group)			*Globularia alypum* aqueous extract decreases hypertriglyceridemia and ameliorates the oxidative status of the muscle, kidney, and heart in rats fed a high-fructose diet.	2	
Karuna 2011^[^^13^^]^	India	Oxidative stress and insulin resistance In fructose-fed rats, hepatic antioxidants and oxidative stress were observed.	Yes	60 days	Oral-fed with fructose diet	High-fructose (66%) diet	Male rats (Albino Wistar)		Yes		Four groups (eight per group)			Preventive effect of *Catharanthus roseus* (Linn.) against high-fructose diet-induced insulin resistance and oxidative stress in male Wistar rats	3	
Hsu 2015^[^^14^^]^	USA	Neurological and memory disorders Consumption of extra sugars in the diet, especially HFCS-55, has a negative effect on hippocampal function, metabolism reactions and neuro-inflammation in case of high-dose intake of fructose during puberty and growth \	Yes	30 days	Oral	HFCS-55 solution, prepared to contain equal carbohydrate weight/volume (11%)	Male rats (Sprague-Dawley)		Yes		38 dolescents + 38 adults (12-13 per group)			Effects of sucrose and HFCS consumption on spatial memory function and hippocampal neuroinflammation in adolescent rats	4	
García-Berumen 2019^[^^15^^]^	Mexico	Effect on the liver Fructose-induced liver damage was related to the extent of dysfunction and oxidative damage in mitochondria.	Yes	6 weeks	Oral	with 25% fructose in the drinking water		Male rats (Wistar)		Yes		Four groups (four per group)	The severity of rat liver injury by fructose and high fat depends on the degree of respiratory dysfunction and oxidative stress induced in mitochondria		5
Mock 2017^[^^16^^]^	USA	Effect on hepatic lipid metabolism Excessive consumption of sweetened beverages and the results of gene expression and analysis of fatty acid compounds showed that some sugars were more harmful to the liver in the hypercaloric state. Consumption of HFCS-55 should be limited.	Yes	8 weeks	Oral	Water sweetened with 13% (w/v) HFCS-55		Female rats		Yes		Four groups (seven per group)	HFCS-55 consumption alters hepatic lipid metabolism and promotes triglyceride accumulation		6
Collison 2010^[^^17^^]^	Saudi Arabia	Effect on liver This study suggests that HFCS may affect hepatic steatosis.	Yes	Between 6 weeks of age until 32 weeks of age	Oral drinking water	20% HFCS		Female mice		Yes		Four groups (20 per group)	Effect of Dietary Monosodium Glutamate on HFCS-Induced Hepatic Steatosis: Expression Profiles in the Liver and Visceral Fat		7
Zhang 2012^[^^18^^]^	China	Effect on the liver Chronic fructose intake can trigger liver stress, which may be important in fructose-induced hepatitis.	Yes	8 weeks	Oral	Water containing 30% fructose		Female mice (CD-1)		Yes		22 mice (12 per group)	Endoplasmic reticulum stress is involved in hepatic SREBP-1 c activation and lipid accumulation in fructose-fed mice		8

**Study characteristics**

Eight studies, including experimental studies, were included in the systematic review. The selected articles were published between 2010 and 2019. In all the studies, rodents, including rats (male and female) and female mice, were examined.

**Molecular analyses of high-fructose consumption in the liver**

***Male rats***

Based on our search strategy, we found five studies in which molecular analyses were conducted on the liver of male rats. A study by Cioffi et al.^[^^11^^]^ showed that a fructose-rich diet significantly changed the levels of plasma 8-OHdG and CAT protein in the mitochondria of rat liver compared to the control rats; 8-OHdG was used as an indicator of oxidative DNA damage in vivo and in vitro. According to the above study, rats fed a high-fructose diet had a higher number of mtDNA lesions and an increasing mtDNA damage in their liver. Also, researchers observed a reduction in mtDNA copy number, indicating impaired mitochondrial biogenesis. Their findings revealed that a diet rich in fructose can cause mtDNA damage and affect mitochondria, which leads to liver dysfunction and metabolic diseases^[^^11^^]^. According to a study performed by Taleb-Dida et al.^[^^12^^]^, CAT activity was not a sensitive test in the studied tissues, except for the liver, after consumption of a high-fructose diet for 14 weeks. However, high levels of TBARS and decreased SOD and CAT activities were found in the liver, which indicate an elevated oxidative stress in rats fed a high-fructose diet. Our findings suggest that fructose overfeeding can induce pro-oxidant effects by increasing TBARS levels and decreasing SOD and CAT activity in the liver. In Karuna and Saralakumari’s^[^^13^^]^ study, reduced antioxidant enzymes activities decreased hepatic GSH levels and increased lipid peroxidation intermediates in the group fed fructose, which result in the development of oxidative stress in high-fructose diet-fed rats for 60 days. Moreover, rats in the fructose group showed significantly higher levels of TBARS and protein carbonyl groups and lower levels of hepatic GSH compared to the control rats. They also exhibited that the activities of antioxidants enzymes, particularly GR, GPx, GST, SOD, and CAT, were significantly lower in the fructose group rats than in the control group. Hsu et al.^[^^14^^]^ found that the exposure of adolescent rats to HFCS-55 within 30 days increased circulating insulin levels and hepatic proinflammatory cytokine expression. Overall, the data of their study showed significant side effects for excessive consumption of sugars, particularly HFCS-55, during critical periods of development, as well as highlighted that metabolic disruptions could occur in adolescent who consume HFCS-55 and sucrose^[^^14^^]^. In a study performed by García-Berumen et al.^[^^15^^]^, mitochondrial lipid peroxidation was exacerbated in the fructose-fed group. The group showed a low level of steatosis and no changes in ROS production, but high levels of mitochondrial lipid peroxidation. This observation shows that mitochondrial lipid peroxidation alone may not cause severe liver damage in the absence of other factors such as significant accumulation of fat or increased mitochondrial ROS production. The study also highlighted that induction of lipid peroxidation in the liver by fructose is linked to the depletion of antioxidant defenses^[^^15^^]^. Despite producing high levels of lipid peroxidation, fructose had less deleterious effects on mitochondrial function and development of NAFLD. This reaction is because fructose partially decreases oxidative phosphorylation and induces a lower percentage of microvesicular steatosis, which can help protect it against the development of NAFLD. It is worth noting that the combination of a high-fat and high-fructose diet has more deleterious effects on mitochondria, including lipid peroxidation, increased ROS production, decreased complex I activity, and the full inhibition of oxidative phosphorylation. These findings are consistent with the severe liver damage induced by this diet^[^^15^^]^.

***Female rats***

Following our search, we found that only one study has examined the effect of high-fructose consumption on liver in female rats. According to Mock et al.^[^^16^^]^, rats that drank HFCS-55 had a significantly higher liver content of MUFA palmitoleic acid compared to those drinking sucrose and fructose solution. Additionally, rats drinking HFCS-55 solution had the highest hepatic content of MUFA palmitoleic acid among all the groups studied, which highlights the unique metabolic effects of HFCS-55 on the liver. PPARα gene expression was downregulated in rats drinking HFCS-55 solution, as compared to the rats drinking water. The desaturation indices indicates a higher SCD-1 activity in rats consumed the HFCS-55 solution than the control group. Rats consumed HFCS-55 solution exhibited a pronounced hepatic de novo lipogenesis, as indicated by the upregulation of stearoyl-CoA desaturase-1 and increased oleic acid content. Such increased lipogenesis was accompanied by a decrease in β-oxidation, which is evident from the downregulation of hepatic peroxisome proliferator-activated receptor α. Researchers attributed the observed lipogenic effects in rats to the slightly higher fructose content of the HFCS-55 solution, despite that there is no difference in macronutrient and total caloric intake between the rats consumed HFCS-55 and those consumed sucrose solution. Results from gene expression and fatty acid composition analysis showed that in a hypercaloric state, certain types of sugars are more detrimental to the liver. Results of this preclinical study showed that the excess consumption of caloric sweetened beverages, particularly those sweetened with HFCS, should be limited^[^^16^^]^.

***Female mice***

Based on our study strategy, we identified two studies investigating the hepatic effects of high fructose consumption on female mice. Collison et al.^[^^17^^]^ compared the gene expression profile in the liver and abdominal fat of animals fed with isocaloric standard chow under identical conditions with that of control animals. The consumption of HFCS resulted in hepatic steatosis and a significant increase in gene expression related to carbohydrate and lipid metabolism. The most significant impact on hepatic gene expression profile was observed in the mice fed with the HFCS diet. Enzymes involved in steroid and fatty acid metabolism from the cyp450 family, including oxidoreductases Cyp2b9 and Cyp2b10, were upregulated by HFCS. Levels of other oxidoreductases, Alox3 and Alox8, involving in inflammatory lipid metabolism, were also found to be increased by HFCS. Additionally, the HFCS diet induced the expression of several inflammatory mediators, including tumor necrosis factor (TNF-α) and IL‐1. Rats that consumed the HFCS solution also showed an increase in endoplasmic reticulum stress-associated PDIA4. Levels of the genes involved in the apoptotic pathway, including G0s2 (the G0/G1 switch gene) and Tnfrsf10b (death receptor), were also upregulated in the HFCS‐diet group. These data suggest that dietary HFCS causes an increase in the TG content of the liver by a mechanism that upregulates genes contributing to carbohydrate and lipid metabolism^[^^17^^]^. According to Zhang et al.^[^^18^^]^, consumption of fructose solution resulted in the activation of hepatic SREBP-1c in mice, in which two target genes of SREBP-1c, fas and acc, were upregulated. The activation of hepatic SREBP-1c by fructose was associated with the depletion of Insig-1^[^^18^^]^. An ER stress and UPR, as determined by an increased GRP78 expression and eIF2α and PERK phosphorylation, were observed in the livers of mice fed with a fructose solution. Altogether, ER stress contributes, at least in part, to hepatic SREBP-1c activation and lipid accumulation in fructose-evoked NAFLD^[^^18^^]^. The data collected from these studies are represented in Tables 2 and 3. 

## DISCUSSION AND CONCLUSION

In this systematic review, we investigated different studies evaluating the effects of fructose consumption on liver damage. Finally, we selected eight animal-based (rodent) studies, five (62.5%) used a male rat model, whereas one (12.5%) and two (25%) used female rats and mice, respectively. This study was conducted to provide better understanding of the molecular mechanism and expression profile of genes involving in the liver damage and caused by fructose in rodent diets.

Although mitochondrial damage and metabolic disorders are predominantly observed in males, females have been found to exhibit an increased hepatic lipogenesis. However, it is notable that both males and females indicate augmented expression of pro-inflammatory cytokines and hepatic inflammatory mediators^[^^14^^,^^17^^]^. Inflammatory cytokines are signaling molecules secreted by helper T lymphocytes and macrophages, intensifying inflammation and increasing inflammatory reactions. These molecules, including IL-1, IL-12, IL-18, TNF-α, interferon gamma, and granulocyte-macrophage colony-stimulating factor, play a crucial role in the innate immune system^[^^19^^,^^20^^,^^21^^]^. Studies have shown that feeding rodents with fructose can increase these factors. Han et al.^[^^22^^]^ reported the elevated levels of IL1, IL-6, and TNF-α in the high fructose-fed male mice. In another study, Al-Qahtani et al.^[^^23^^]^ found potential gender differences in lipid kinetics. Their study suggested that disparities in energy sensing between males and females could be a reason for the less noticeable metabolic changes in the livers of female rats caused by fructose.

As represented in Table 3, the levels of TBARS in rat liver increased with a high-fructose diet, whereas activities of antioxidants enzymes (GR, GPx, GST, SOD, and CAT) decreased^[^^12^^,^^13^^]^. According to a study by Busserolles et al.^[^^24^^]^, rats fed with a fructose diet showed higher levels of TBARS in plasma and urine samples than those fed with a starch diet^[^^24^^]^. Also, lipid peroxidation was found to be increased in samples obtained from patients with metabolic syndrome. Male rats fed with HFCS-55 showed metabolic disruptions, while female rats exhibited lipogenic effects on the liver^[^^14^^,^^16^^]^. Also, in mice models, lipogenic gene expression increased when fed with HFCS^[^^17^^,^^25^^]^.

One of the major outcomes of a high-fructose diet is a raise in lipid peroxidation^[^^13^^,^^15^^]^. Lipid accumulation in fructose-evoked NAFLD was observed in the mice fed a fructose solution^[^^18^^]^. The rats that were fed with HFCS-55 solution showed a variety of outcomes and alterations in function, such as changes in the levels of plasma 8-OHdG and CAT protein, mitochondrial biogenesis, liver dysfunction, metabolic diseases, and fat oxidation.^[^^11^^,^^14^^,^^17^^]^. Oxidative stress was observed as a consequence of chronic fructose feeding in rats^[^^11^^,^^12^^,^^26^^]^.

**Table 2:** Effects of fructose consumption in molecular analyses of fructose-fed animals

**Duration of study/treatment**	**The most important outcomes of molecular analyses**	**Species/gender of animals**	**Authors/** **date**
8 weeks	Rats fed a fructose-rich diet had significantly higher levels of plasma 8-OHdG compared to the control group. CAT protein levels in rat liver mitochondria decreased, while liver mtDNA contained significantly more mtDNA lesions.	**Male** **rats**	Cioffi 2017^[^^11^^]^
14 weeks	In the fructose diet group, compared to the control group, SOD activity decreased in the liver, and heart and inversely increased in adipose tissue. GPx activity reduced in the heart and increased in adipose tissue, whereas the values remained unchanged in the liver, muscle, and kidney. Reduced CAT activity was noted.	Male rats	Taleb-Dida 2011^[^^12^^]^
60 days	Concerning liver antioxidant status in fructose-fed rats, oxidative stress was observed.	Male rats	Karuna 2011^[^^13^^]^
30 days	Protein expression of pro‐inflammatory cytokines (IL-6 and IL-1β) increased in the dorsal hippocampus for the adolescent HFCS‐55 group. Liver IL-1β and plasma insulin levels elevated in adolescent‐exposed HFCS-55 groups.	Male rats	Hsu 2015^[^^14^^]^
6 weeks	In the fructose group, the exacerbation of mitochondrial lipid peroxidation was observed.	Male rats	García-Berumen 2019^[^^15^^]^
8 weeks	REBP-1c was upregulated in the fructose group. Gene expression of PPARα was downregulated in the HFCS-55 group compared to control.	Female rats	Mock 2017^[^^16^^]^
26 weeks (between 6 weeks of age until 32 weeks of age)	Expression of carbohydrate and lipid metabolism genes was induced.	Female mice	Collison 2010^[^^17^^]^
8 weeks	Fructose stimulation led to the activation of hepatic SREBP-1c, which is linked with the depletion of Insig-1. Additionally, noticeable ER stress and an UPR were observed in the liver.	Female mice	\ Zhang 2012^[^^18^^]^

It has also been observed that metabolic diseases can be triggered by chronic inflammation and oxidative stress, which are closely associated with a high-fat or high-carbohydrate diet. Such diets can lead to lipid peroxidation and protein carbonylation, as well as a decline in the body's antioxidant content and glutathione levels^[^^27^^]^. Studies have shown that a fructose-rich diet can damage the liver, reduce antioxidant capacity and cause oxidative damage^[^^28^^,^^29^^]^. Researchers have also suggested that a high-fructose diet may contribute to the development of metabolic syndrome, including hepatic steatosis^[^^30^^,^^31^^]^. Hepatocytes have a complex network of nonenzymatic and enzymatic antioxidant defenses designed to eliminate or counteract ROS. This sophisticated system protects the liver from oxidative stress and maintains its proper function. However, in the presence of the excessive levels of ROS, it can overwhelm the hepatocellular antioxidant defenses, which results in hepatocyte injury, ROS production, and cell death^[^^32^^]^. 

The 8-OHdG is a great base modification in mammalian DNA, and its level is directly related to oxidative stress. Therefore, it can be used as a biomarker for systemic oxidative stress in in vivo conditions^[^^11^^,^^33^^-^^35^^]^. The results of studies included in our review showed reduced CAT expression in rats fed with a high- fructose diet, which is consistent with previous studies showing an increase in oxidative stress in the hepatic mitochondria of rats fed with a fructose-rich diet^[^^11^^,^^28^^]^. A review included the results of the studies relating to the effects of a fructose-rich diet on mitochondria, in which they have shown significant mtDNA, indicating that the long-term consumption of high fructose diet affects mitochondria and possibly causes metabolic disorders such as insulin resistance and hepatic steatosis^[^^11^^,^^36^^,^^37^^]^. Based on the above-mentioned studies, lipid peroxidation can be considered as an extractor of nonalcoholic steato-hepatitis by triggering signaling cascades, which mediate inflammation via increasing in the level of malondialdehyde and 4-hydroxynonenal, the end- products of lipid peroxidation, suggesting that lipid peroxidation induced by fructose may trigger inflammation^[^^15^^,^^38^^,^^39^^]^. An earlier study conducted on mice has found that fructose induces hepatic lipid accumulation, activates hepatic SREBP-1c, causes hepatic ER stress and decreases hepatic Insig-1 protein^[^^18^^]^. A fructose-rich diet negatively affects liver function and metabolism^[^^32^^]^ because almost all the absorbed monosaccharides are primarily sent to the liver via portal blood, and approximately all ingested fructose is metabolized in the liver^[^^11^^,^^40^^,^^41^^]^. Accordingly, the massive flow of ingested fructose and its handling in the liver cause a metabolic injury to the tissue, which is well-established^[^^11^^,^^42^^-^^44^^]^. 

**Table 3:** Main molecular analysis outcomes of fructose in the liver of rat and mice

**No.**	**Alteration/dysfunction**	**Decrease/reduction**	**Increase/enhancing/ inducing**	**Target organ**		**Type of fructose**	**Author/date**
1	Changed levels of plasma 8-OHdG	mtDNA copy number	mtDNA damage	Rat liver mitochondria		HFCS-55	Cioffi 2017^[^^11^^]^
	Changed CAT protein levels						
	Mitochondrial biogenesis		Plasma 8-OHdG				
	Liver dysfunction and metabolic diseases		Oxidative stress				
2	CAT activity	SOD and CAT activities	Levels of TBARS	Rat liver		High-fructose diet	Taleb-Dida 2011^[^^12^^]^
			Oxidative stress				
3	-------	Activities of enzymatic antioxidants GR, GPx, GST, SOD, and CAT	Levels of TBARS Protein carbonyl groups Lipid peroxidation intermediates	Rat liver		High fructose diet fed	Karuna 2011^[^^13^^]^
		Levels of hepatic GSH					
4	Metabolic disruptions	------	Circulating insulin levels	(Liver) adolescent rats		HFCS-55	Hsu 2015^[^^14^^]^
			Hepatic proinflammatory cytokine expression				
5	-------	Level of steatosis	Mitochondrial lipid peroxidation	Male Rat liver		Fructose-fed group	García-Berumen 2019^[^^15^^]^ .
		Oxidative phosphorylation					
6	Lipogenic effects	PPARα gene expression (downregulated)	Liver content of the MUFA, palmitoleic acid		Female rat liver	drinking HFCS-55	Mock 2017^[^^16^^]^
			Hepatic content of oleic acid				
			SCD-1 (stearoyl-CoA desaturase-1) activity				
			Hepatic de novo lipogenesis				
			Expression of genes involved in carbohydrate and lipid metabolism				
			Oxidoreductases Cyp2b9 and Cyp2b10 (upregulated)				
			Oxidoreductases Alox3 and Alox8				
			The expression of TNF-α and IL‐1				
			Endoplasmic reticulum stress‐associated Pdia4				
			Levels of genes involved in the apoptotic pathway: G0s2, G0/G1, Tnfrsf10b (upregulated)				
			The TG content of the liver				
			The expression of lipogenic genes				
8	ER stress and UPR	Insulin-induced gene (Insig)-1	Hepatic SREBP-1c		Mice liver	fructose solution	Zhang 2012^[^^18^^]^
			fas and acc				
	lipid accumulation in fructose-evoked NAFLD		GRP78 expression				
			eIF2α and PERK phosphorylation				

Based on the information provided, it seems that the research on the chronic and long-term effects of fructose consumption on rodent models is somewhat limited due to the small number of high-quality studies eligible for our criteria. This limitation do not allow to fully explain the relationships and molecular signalings associated with the consumption of both dietary high fructose and HFCS-55 solution on the liver of rodent models. Hence, high-quality studies are needed to better understand these relationships and molecular signalings. Overall, the results of this systematic review suggest that long-term fructose consumption may cause molecular damage to the liver in animal models.

### Limitations

The present study investigated the effects of fructose consumption on the liver function of rodents, which is a limitation of our study. Examining the effects of a high-fructose diet on humans may provide better insight into the cause-and-effect relationships between high fructose intake and development of liver injury. However, further research is needed to fully understand the implications of fructose for human health. Hence, this issue can serve as a basis for future studies.

## DECLARATIONS

### Ethical statement

Not applicable. 

### Data availability

The data supporting the article are also included within the article and supplementary file. Additionally, the analyzed data sets generated during the study can be made available from the corresponding author upon reasonable request. 

### Author contributions

RM: data curation, formal analysis, investigation, review and editing, writing original draft, methodology, and conceptualization. R.K: data curation, formal analysis, investigation, review and editing, and methodology. SAB: data curation, formal analysis, Investigation, review and editing, methodology, conceptualization, supervision, and project administration. 

### Conflict of interest

None declared.

### Funding/support

There is no funding supported this project.

## Supplementary Materials


