# Supplementary material for: The Role of High-Fructose Diet in Liver Function of Rodent Models: A Systematic Review of Molecular Analysis
Source: Iran Biomed J. 2023 Jul 29;27(6):326–39. doi: 10.52547/ibj.3965 (PMC10826909; doi:10.52547/ibj.3965)
Supplement: Supplementary file 1 [file ibj-27-326-s001.pdf]

Supplementary Table 1. Main databases and Search Strategy

Databases	Search strategy/ keywords
Web of Science, PubMed, Scopus, Embase	"High Fructose Corn Syrup", "HFCS", "High-Fructose Maize", "Fructose", "Glucose Fructose Syrup", "Sweetening", "liver dysfunction", "liver injury", "hepatitis", and "rodent and animal model"<br><br>("HFCS" OR "High-Fructose Maize Syrup" OR "High Fructose Maize Syrup" OR "Corn Syrup" OR "Corn Sugar" OR "Maize Syrup" OR "Maize Syrup", "High-Fructose" OR "Syrup, High-Fructose Maize" OR "Fructose" OR "Glucose" OR "Glucose Fructose Syrup" OR "Sugars" OR "Agent", "Sweetening, OR Agents", "Sweetening" OR "Sweetening Agent" OR "Sweeteners" OR "Sweetener" OR "Sugar Substitutes" OR "Substitute", "Sugar" OR "Substitutes, Sugar OR Sugar Substitute OR Artificial Sweeteners" OR "Artificial Sweetener" OR "Sweetener", "Artificial" OR "Sweeteners", Artificial , OR "sugar-sweetened beverages" OR "soft drink" OR "soft drinks" OR "beverage" OR "beverages")<br><br>AND ("Lipid metabolism" OR "Obesity" OR "Fat accumulation" OR "Diabetes" OR "Liver" OR "Liver Dysfunction" OR "Liver fat" OR "Fatty Liver" OR "Fatty Acids" OR "NAFLD" OR "Nonalcoholic fatty liver disease" OR "Liver Disease" OR "Mitochondrial" OR "Mitochondrial activities" OR "Cancer" OR "Hormonal Dysregulation" OR "Molecular analysis" OR "Gene Expression" OR "Protein Expression" OR "Enzymes" OR "Oxidative Enzymes" OR "Precocious Puberty" OR "Puberty OR "Hyperlipidemia" OR "Lipidemia" OR "Insulin Resistance" OR "Steroidogenesis" OR "Oxidative Stress" OR "inflammation")<br><br>NOT ("Medicine", "Therapy", "interventional mixture", "synergistic effects", "corn seed", "mixed diet", "cell culture method", "Epidemiology").
